# Fructooligosaccharide and *Bacillus subtilis* synbiotic combination promoted disease resistance, but not growth performance, is additive in fish

**DOI:** 10.1038/s41598-023-38267-7

**Published:** 2023-07-13

**Authors:** Nilesh Anil Pawar, Chandra Prakash, Mahinder Pal Singh Kohli, Ankur Jamwal, Rishikesh Subhashrao Dalvi, B. Nightingale Devi, Soibam Khogen Singh, Shobha Gupta, Smit Ramesh Lende, Sadanand D. Sontakke, Subodh Gupta, Sanjay Balkrishna Jadhao

**Affiliations:** 1 ICAR-Central Institute of Fisheries Education, Mumbai, 400061 India; 2 ICAR-Central Marine Fisheries Research Institute, Mumbai Centre, Mumbai, 400061 India; 3 Centre for Climate Change & Sustainability, Azim Premji University, Bengaluru, 562125 India; 4 Maharshi Dayanand College (University of Mumbai), Parel, Mumbai, 400012 India; 5 Colleges of Fisheries, Chhattisgarh Kamdhenu University, Raipur, 491995 India; 6 College of Fisheries, Central Agricultural University, Lembucherra, 799210 India; 7 Annasaheb Vartak College (University of Mumbai), Mumbai, 401202 India; 8 Center of Excellence in Aquaculture, Kamdhenu University, Ukai, 394680 India; 9 CSIR-National Environmental Engineering Research Institute, Nagpur, 440 020 India

**Keywords:** Biotechnology, Microbiology

## Abstract

Species diversification from major to minor carps for their sturdiness and initial higher growth, and also a quest for antibiotic-free aqua farming in the subcontinent, mandates search for and evaluation of alternatives. An experiment was performed to investigate the potential of fructooligosaccharide (FOS) and *Bacillus subtilis* (BS) (alone or as synbiotics) in promoting growth and immunity against infections in *Labeo fimbriatus* fingerlings. Six iso-nitrogenous and iso-lipidic diets containing combinations of two levels of FOS (0% and 0.5%) and three levels of BS (0, 10^4^, 10^6^ CFU/g feed) were fed to fish for 60 days. At the end of the feeding trial, twenty-four fish from each group were injected intra-peritoneally with pathogenic strain of *Aeromonas hydrophila* O:18 to test the immunoprotective efficacy of the supplements against bacterial infection. BS, but not FOS, significantly improved (P < 0.05) growth and feed utilisation attributes like percentage weight gain (PWG), specific growth rate (SGR) and feed conversion ratio (FCR). There were interactive effects of FOS and BS on PWG, SGR and FCR; however, the effects were not additive in nature. These beneficial effects of BS, alone or in combination with FOS, were corroborated by increased protease activity, microvilli density and diameter and number of goblet cells. Overall beneficial effects of FOS and BS included improved erythrocyte (RBC), hemoglobin (Hb), total protein and globulin levels. Total leucocyte (WBC) count and immunological parameters like respiratory burst activity of leucocytes (NBT reduction), lysozyme activity, albumin: globulin ratio and post-challenge survival were significantly improved by both FOS and BS, and their dietary combination yielded the highest improvement in these parameters. Synergistic effects of FOS and BS as dietary supplements indicate that a combination of 10^6^ CFU/g BS and 0.5% FOS is optimal to improve growth, feed utilisation, immune functions, and disease resistance in *L. fimbriatus* fingerlings.

## Introduction

Recent technological advancements in aquaculture have substantially increased global fish production. In the midst, aquaculture intensification has also resulted in deterioration of water quality and disease outbreaks, causing losses as per World Bank Group to the tune of US$6 billion per year to the sector^1^. The parasitic and infectious diseases itself cost between US$ 1.05 and US$ 9.58 billion per year to the global aquaculture^2^. Thus, tackling such an adverse situation necessitates the use of several drugs and immune boosters (see reviews^3,4^). Antibiotics have been long used in the fish and shrimp industry as growth promoters or to treat several diseases of bacterial/viral origin^5^. However, citing the sustainability issues from the use of antibiotics, such as the development of antibiotic resistance genes and toxicity to zooplankton and phytoplankton (see reviews^5,6^), the industry needs to pay more attention toward finding safer methods and practices.

For the last few decades, the use of plant-based herbal extracts, beneficial microbes, and other biological compounds have gained immense interest and shown promise as growth promoters and prophylactic agents^7,8^. The application of phage and vaccines are in line; however, they are costly. Instead, use of beneficial bacteria (probiotic) have demonstrated a positive effect on growth, feed efficiency, and health status of aquaculture organism when applied alone or in combination with a suitable probiotic substrate^9–15^. Deliberate manipulation of host microbial consortia composition by synbiotic, “a mixture comprising live microorganisms and substrate(s) selectively utilized by host microorganisms that confers a health benefit on the host”, is a novel approach not only from nutritional point of view, but also as an alternate health promoting tool to overcome the adverse effects of antibiotics and drugs^16,17^. Beneficial effects of a symbiotic relationship is largely attributed to the physiological, biochemical, and antimicrobial action, as well as the capacity of beneficial microorganism to competitively exclude pathogenic population in the intestinal tract of the host organism^18^. However, the potentiality of the probiotic also depends on the host species in concern, its form of application (spore/encapsulated), and the doses administered^19^.

Mono-species or multispecies commercial supplements containing organisms of the *Bacillus* genus have gained wide acceptance in aquaculture due to its spore-forming abilities and resistance to aggressive physical and chemical conditions. Various species of *Bacillus* also demonstrate peculiar physiological features that enables them to survive in diverse environmental conditions, including fresh water, marine sediments, desert sands, and hot springs^20^. The strain of *B. subtilis* (MTCC-121) used in the present study is reported to enhance activity of digestive enzymes and competitively exclude *Acetonobacter* sp., and *Salmonella* sp. in the gut of *M. rosenbergii* post larvae^21^, and stimulate immunity and growth in *L. fimbriatus* fingerlings^22^.

While there is abundance of information on the benefits of probiotics in promoting health of aquatic organisms, application of prebiotic as substrate for probiotic growth in gut is still an evolving area of research. Prebiotics serves as energy sources for the intestinal micro-biota and can modulate immunity through direct interactions with the innate immune system or by proliferation and growth of beneficial micro-biota^23^. Several prebiotics are examined and used in aquaculture such as inulin, oligo-fructose, xylooligosaccharide, fructooligosaccharide (FOS), mannanoligosaccharide (MOS), galactooligosaccharide (GOS) and β-glucan. Existing literature on prebiotics suggest their benefits on growth, survival and feed utilization^22,24–29^, stress mitigation^30^, modulation of immune-gene expression^31^ and disease resistance against viral, bacterial, and fungal attacks^32,33^ in fish. Among the evaluated prebiotics, FOS has been extensively evaluated and reported in cultured fishes^34,35^. During the past decade, the concept of using probiotic and prebiotic in combination to achieve synergistic action has been extensively studied by research groups across the globe. Several synbiotic combinations such as *Enterococcus faecalis* + MOS^36^; *Entercoccus faecium* + FOS^37^; *B. subtilis* + FOS^38^; *B. subtilis* + Chitosan^39^; *B. clausii* + FOS + MOS^40^; *Bacillus* OJ + IMO^41^; *B. licheniformis* + IMO^42^; *Bacillus* spp. + MOS, FOS^22,35,43–45^ have been examined for aqua farming. Synbiotic applications containing *B. subtilis* have been reported earlier in cultivable fishes like fringed lipped carp, *L. fimbriatus*^22^; mrigal, *Cirrhinus mrigala*^46^ and Nile Tilapia, *Oreochromis niloticus*^47^. However, most of the reports have focussed largely on few freshwater and marine species, and needs proper evaluation in other commercially important fishes, especially the medium and minor carps that are in high demand in rural Asian countries and are nutritionally important due to their affordable price.

For many decades, aquaculture in the Indian subcontinent was exclusively dependent on the farming of three Indian Major Carps (IMC) namely, catla (*Catla catla*), rohu (*Labeo rohita*) and mrigal (*C. mrigala*). Recently, the subcontinent region is witnessing significant development in terms of both horizontal and vertical expansion of the carp farming activities. Species diversification, considering local demand and regional climatic adaptability has taken a leap, with the governmental emphasis laid on promoting endemic species for purpose of culture and conservation of wild-stock^48,49^. In the IMC oriented culture system, incorporation levels of the various species of medium and minor carps have been standardised. Owing to initial higher growth rate and market acceptability at 300–400 g, the medium sized carps have been advocated as ideal species for intercropping in the carp farming system^50^. Amongst medium carp, *L. fimbriatus* (fringed lipped carp) is considered as a good choice for aquaculture diversification due to its excellent flavour, meat quality^48,51^, and environmental adaptability during high density seed rearing^52^. The species is more suitable for the utilisation of seasonal ponds, which have 5–6 months of water retention or inclusion as a component of traditional carp polyculture on the basis of their regional significance^50,53,54^.

With an interest to identify safe biological methods of immune stimulation for increase in productivity of selected endemic carps like *L. fimbriatus*, we conducted this study to evaluate (a) the use of a potential probiotic strain *B. subtilis* (MTCC-121) and prebiotic fructooligosaccharide, either individually or in combination, in *L. fimbriatus* growth promotion, (b) the potency of *B. subtilis* (MTCC-121) in boosting the immune and digestive functions, and (c) the protection provided by *B. subtilis* (MTCC-121) against *A. hydrophila* challenge. The results of the study can invariably provide a safe strategy for disease protection of *L. fimbriatus,* which will enhance overall growth and survival in culture ponds.

## Material and methods

### Ethics statement

All the methods were carried out following relevant national and international guidelines and regulations. The handling and care of the experimental fishes was done in compliance with the guidelines of the Committee for the Purpose of Control and Supervision of Experiments on Animals(CPCSEA)^55^. The study protocols and experimental endpoints were further approved by the research advisory committee (RAC) of the Indian Council of Agriculture Research (ICAR)-Central Institute of Fisheries Education (CIFE), Mumbai, India. Proper husbandry and handling practices were executed during the whole experiment and reported to the institute committee. The study complies with the Animal Research: Reporting of in Vivo Experiments (ARRIVE) guidelines.

### Experimental animals and maintenance

The experimental trial was carried out at the aquaculture wet laboratory facility of the ICAR-CIFE, Mumbai, India. A total of 1250 *L. fimbriatus* fry (5.20 ± 0.21 cm length, 2.50 ± 0.16 g weight) were procured from the ICAR-Central Institute of Freshwater Aquaculture (CIFA), Bhubaneswar, India. Upon arrival, the fishes were stocked in rectangular fibre reinforced plastic (FRP) tanks which were previously disinfected and provided with round the clock aeration. As a prophylactic measure, fish were given dip treatment in 5% KMnO_4_ solution for five minutes before stocking. The fish were acclimatized for another 30 days before their use in the experiment. During the acclimation period, fish were fed twice a day with practical diet containing 30% crude protein at the rate of 5% of body weight, split into two equal portions. At the end of the acclimation period, and beginning of the experiment, fish reached an average size of 6.65 ± 0.16 cm with an average weight of 3.85 ± 0.18 g.

### Experimental systems

Experiments were carried out in uniform sized rectangular plastic tubs (80 × 57 × 42 cm, 150 L capacity, Nilkamal Ltd., Mumbai, India) covered with perforated lids. Before initiation of the experiment, tubs were washed and filled with KMnO_4_ solution (4 ppm) and left overnight. Tanks were flushed and thoroughly washed with chlorine-free water on the following day. Total volume of the water in each tub was maintained at 100 L throughout the experimental period (evaporation loss refilled whenever required). Round-the-clock aeration was provided through a centralised air blower connected to the individual tanks. Water quality parameters namely dissolved oxygen (DO), temperature, pH, free CO_2_, total hardness, ammonia, nitrite and nitrate were monitored and recorded following standard procedures^56^. During the experimental period, the water parameters examined were within the optimum range (see Table S1, Supplementary) for culture of carps. The free CO_2_ was not detectable during entire experimental period as constant air supply was maintained in the experimental tanks.

### Experimental design

Out of the total stock, a total of 270 uniformly sized healthy fingerlings (3.94 ± 0.34 g; avg. weight) were randomly distributed into eighteen FRP tanks, with fifteen fish in each tank. The tanks were randomised into six distinct groups, corresponding to six different dietary treatments, in a 2 × 3 factorial design involving two levels of FOS and three levels of BS (*B. subtilis*). Each treatment was conducted in a triplicate manner. The six treatment groups were **Control**: No FOS and No BS, **FOS:** 0.5% FOS and no BS, Low BS (**LBS**): 1 × 10^4^ colony forming units (CFU) of BS and no FOS, **FOS + LBS:** 0.5% FOS and 1 × 10^4^ CFU of BS, High BS (**HBS**): 1 × 10^6^ CFU of BS and no FOS, and **FOS + HBS:** 0.5% FOS and BS at 1 × 10^6^ CFU**.**

### Bacterial culture

A lyophilized form of *B. subtilis* (MTCC-121) was sourced from the Microbial Type Culture Collection and Gene Bank (MTCC), Chandigarh, India. Prior to mass culture, the viability and growth of the strain in prebiotic (FOS) was evaluated in 96-well microtitre plates. The bacteria were grown in nutrient broth (HiMedia) under aerobic conditions at 30 °C for 24 h in a mechanical shaker incubator at 150 rpm (SI505, Stuart, UK). Subsequently, a loopful of the bacterial culture was streaked on nutrient agar (HiMedia) plate. The bacterial colonies, which grew on the nutrient agar, were re-confirmed as pure isolates of *B. subtilis* by performing the essential micro-observation and biochemical tests (HiBacillus ™ Identification Kit, Himedia, Mumbai, India), and were mass cultured for subsequent use in the experiment. The bacterial culture was maintained by transferring bacterial cells to fresh nutrient broth after 48 h. For incorporation in the diet, the culture was centrifuged at 10,000*g* for 20 min at 4 °C and the supernatant was discarded, whilst the pellet was re-suspended in PBS (pH 7.2). The suspension was similarly washed and re-centrifuged three times and then quantified by the spread plate technique (nutrient agar, incubated at 30 °C for 24 h). The suspension of the probiont was diluted to the requisite levels with PBS and incorporated in 100 g feed. Purified and quantified bacteria were kept in suspended form at 4 °C and were used as and when required for feed preparation.

### Bacterial quantification

To determine the concentrations of the bacterial inoculums to be added into the feed, the probiotic strain was streaked on nutrient agar plates and incubated for 12 h at 30 °C. One colony was transferred to 50 ml of nutrient broth and incubated under the same conditions for 4 h. A third transfer for bacteria was carried out into 100 ml, under same conditions. Then optical density (O.D.) of the bacterial samples was recorded at 600 nm. The dilutions were plated onto the respective agar by spread plate technique. After 12 h of incubation at 28 °C, colonies were counted using a colony counter (Suntex, Taiwan). The data plot obtained for relationship of CFU versus OD_600_ versus time was used for quantification and accordingly added to the test diets to achieve the predetermined concentration of 10^4^ or 10^6^ CFU/g.

### Preparation of experimental diets

A basal diet was formulated using purified ingredients for the study (Table 1). Six iso-nitrogenous and iso-lipidic diets were prepared by incorporating the required count of *Bacillus subtilis* (BS) (0, 1 × 10^4^ or 1 × 10^6^ CFU g^−1^ diet) and/or fructooligosaccharide, FOS (0 or 0.5%) (Raftilose P95; DPO Foods, Thane, India) (see Fig. S1: Supplementary) to the basal diet. Amount of cellulose, equivalent to 0.5% FOS, was added to the diets that received 0% FOS. Required amount of ingredients were weighed and mixed uniformly. Prebiotic was mixed in chilled distilled water and blended with the mixed ingredients. To the mixture, measured volume of oil and water, if necessary, were also added to form a dough which was passed through the pelletizer. A single-screw hand pelletizer with a 1 mm die diameter was used to prepare the diets. The probiotic culture (suspended in PBS) prepared previously was sprayed over the wet pellets. The diet of the control group was sprayed with sterile solution of phosphate buffer (pH 7.4). The pellets were left for 24 h to dry at room temperature (26–28 °C). After proper drying, the diets were packed in air-tight plastic pouches and stored at − 20 °C until used. Following storage, viable count in the feed was checked for four weeks following standard methods^57,58^.

**Table 1:** Composition of the basal diet.

Ingredient	Percent
Casein^a^	35
Dextrin^b^	10
Cellulose^b^	08
Starch^b^	25
Gelatin^c^	08
Cod liver oil	05
Sunflower oil	03
Carboxymethyl Cellulose(CMC)^b^	02
Vitamin-Mineral premix^d^	03
Betaine hydrochloride^b^	01
Total	100
Betahydroxytoulene (BHT)	0.01
Crude protein (CP)	35.0
Ether extract (EE)	7.00
*DE (kcal kg^−1^)	3550

^a,b^^,c^Purchased from HiMedia Ltd., India.

^a^Fat free form with 75% CP.

^c^Contains: 96% CP.

^d^For composition of premixused in our lab, see Gupta. et al.^44^.

*Digestible energy (kcal kg^−1^) = (% CP × 4) + (%EE × 9) + (NFE × 4) (as per AOAC method).

Experimental diets were devised from the above basal (control) diet by substituting equivalent amount of cellulose with fructoligosaccharide, FOS (0, 0.50%) and three levels of *Bacillus subtilis* (BS) (0, 1 × 10^4^ and 1 × 10^6^ CFU g^−1^ diet) to form six diets as: Control: Without FOS/ BS, FOS (0.5% FOS alone), Low BS(LBS): BS at 1 × 10^4^ alone, FOS + LBS, High BS (HBS): BS at 1 × 10^6^ alone and FOS + HBS.

### Experimental feeding

Prepared test diets were fed to triplicate groups of fish twice daily (09:00 h and 17:00 h) for 60 days. Initially, feeding was done at 4% of body weight and gradually adjusted to 3% in the later stage of the experiment based on fortnightly growth data. Uneaten feed and faecal matter were siphoned out daily and the same volume of water was replaced from the storage tank.

### Sampling procedure

Growth performance of *L. fimbriatus* fingerlings fed different experimental diets were assessed for percentage weight gain (PWG), specific growth rate (SGR) and food conversion ratio (FCR). The fishes were starved for 24 h before the final sampling. The PWG, SGR and FCR were evaluated based on the standard formulae given below:

$${\text{Percentage weight gain }}\left( {{\text{PWG}}} \right) \, = \, \left( {{\text{final weight }}{-}{\text{ initial weight}}} \right)/\left( {\text{initial weight}} \right) \times {1}00$$

$${\text{Specific growth rate }}\left( {{\text{SGR}}} \right) \, = {1}00 \, \left[ {{\text{log}}_{{\text{e}}} \left( {{\text{average final weight }} - {\text{ average initial weight}}} \right)} \right]{\text{/number of culture days}}$$

$${\text{Feed conversion ratio }}\left( {{\text{FCR}}} \right) \, = {\text{ total dry feed intake }}\left( {\text{g}} \right){\text{/wet weight gain }}\left( {\text{g}} \right)$$

$${\text{Feed conversion ratio }}\left( {{\text{FCR}}} \right) \, = {\text{ total dry feed intake }}\left( {\text{g}} \right)/{\text{wet weight gain }}\left( {\text{g}} \right)$$

$${\text{Feed efficiency ratio }}\left( {{\text{FER}}} \right) \, = {\text{wet weight gain }}\left( {\text{g}} \right)/{\text{dry feed fed }}\left( {\text{g}} \right)$$

At the end of the feeding trials, fish from different treatment groups were euthanized after anesthetization with clove oil (50 μL/L) and different tissues were immediately dissected, weighed and kept at − 20 °C for enzymatic assays. For immune assays, blood was drawn from the caudal vein using an insulin syringe (24 gauge) that was previously rinsed with ethylene diamine tetra-acetic acid (EDTA) disodium salt (2.7%). The blood was immediately transferred to a 2 ml centrifuge tube containing dried EDTA to prevent clotting. For collection of serum, blood was drawn without the use of anticoagulant and allowed to clot for 2 h, centrifuged at 5000×*g* to collect a straw-coloured serum that was immediately stored at **− **20 °C for further analysis.

### Challenge study

After 60 days of feeding trial, 24 fish from each experimental group were injected intra-peritoneally with pathogenic strain of *A. hydrophila* O:18 procured from the bacterial culture collection facility of AAH Division, ICAR-CIFE, Mumbai, India. *A. hydrophila* was grown on nutrient broth (HiMedia Ltd, Mumbai, India) for 24 h at 30 °C. The culture broth was centrifuged at 3000×*g* for 10 min to obtain a pellet of bacterial cells that was re-suspended in sterile phosphate buffer saline (PBS, pH 7.4). The final bacterial concentration was adjusted to 1.0 × 10^6^ CFU/mL by serial dilution method. Fish mortality was observed, post bacterial injection, for all the treatment groups for 7 days. The number of surviving fish post challenge were noted daily for 10 days. Dead fish were immediately removed and mortality due to *A. hydrophila* was confirmed after re-isolating it from the dead fish. Survival was calculated using the following formula:

$${\text{Percentage survival }} = \, \left( {{\text{Number of surviving fish after challenge}}/{\text{Number of fish injected with bacteria}}} \right) \times {1}00.$$

The relative level of protection (RLP) of the challenged fish was calculated as per the equation^59^:

$${\text{RLP }}\left( \% \right) \, = \, \left[ {{1 } - \, \left( {{\text{mortality }}\left( \% \right){\text{ of treated group}}/{\text{mortality }}\left( \% \right){\text{ of control group}}} \right)} \right] \, \times { 1}00.$$

### Haematological parameters

Total RBC and WBC count were determined in a Neuberger’s haemocytometer (Feinoptik, Blakenburg, Germany) using erythrocyte and leucocyte diluting fluids (Toission's and Turk’s solution; Qualigens, India), respectively. The following formula was used to calculate the number of erythrocytes and leucocytes per ml of the blood sample:

$${\text{Number of cells}}/{\text{mL }} = \, ({\text{Number of cells counted}} \times {\text{dilution}})/({\text{Area counted}} \times {\text{depth of fluid}}).$$

The haemoglobin percentage was determined by estimating cyanmethemoglobin using Drabkin’s fluid (Qualigens, India) and absorbance (540 nm) was measured using a spectrophotometer (MERCK, Nicolet, evolution 100). The final concentration was calculated by comparing with standard cyanmethemoglobin (Qualigens, India). Blood hematocrit value was measured using standard micro-hematocrit method^60^ and expressed as percentage (%).

### Blood glucose, lysozyme and respiratory burst activity

Glucose was estimated by the method of Somogyi^61^. The respiratory burst activity of the phagocytes was calculated using the reduction of NBT to formazan as a measure of the production of reactive oxygen species (ROS), also called NBT assay, following the method of Secombes^62^ with modification made by Stasiack and Bauman^63^. The OD of the torsique blue coloured solution was then read at 540 nm in an ELISA plate reader (BioRad, USA).

Lysozyme activity was measured following the turbidity assay^64^ using *Micrococcus lysodeikticus* (Sigma, 0.2 mg mL^−1^) suspended (1:20 dilution) in a 0.05 M sodium phosphate buffer (pH 6.2). The reaction was run at 25 °C and absorbance measured at 540 nm (after 0.5 and 4.5 min) in a spectrophotometer. A unit of the activity was determined as the amount of enzyme catalyzing a decrease in absorbance at 540 nm of 0.001/min.

### Serum total protein, albumin (A), globulin (G), A/G ratio

Serum protein was estimated by the Biuret and BCG dye binding method^65^ using commercial kit (total protein and albumin kit, Qualigens Diagnostics, Glaxo Smithkline). Albumin was estimated by the bromocresol green binding method^66^. The absorbance of the standard and test were measured against a blank in a spectrophotometer (Shimadzu, UV1800, Kyoto, Japan) at 630 nm. Globulin was calculated by subtracting the albumin values from the total serum protein. A/G ratio was calculated by dividing albumin values by globulin values.

### Digestive enzyme assays

Tissues were collected and homogenised in chilled sucrose solution (0.25 M) using a Teflon-coated mechanical tissue homogenizer (Remi laboratory instruments, Mumbai). Protease activity was determined by the casein digestion method as described by Drapeau^67^ (Drapeau, 1974). One unit of enzyme activity was defined as the amount of enzyme required to release acid soluble fragments equivalent to 0.001 A_280_ per minute at 37 °C and pH 7.8. The amylase activity on carbohydrates was estimated using di-nitro salicylic acid (DNSA) method^68^ and expressed as mmole of maltose released from starch per min at 37 °C.

### Microscopy

The intestinal tissues from different experimental groups were prepared for histological examination by light and electron microscopy. Tissues for light microscopy were fixed in neutral buffered formalin, embedded in paraffin wax, cut at 5 µm and stained with haematoxylin and eosin (H & E) as described earlier^69^. Tissue sections were examined under a binocular research microscope (Olympus, Japan).

Scanning electron microscopy (SEM) of intestine of selected fish from the experimental groups was carried out using standard procedure^70^. Briefly, tissue was fixed in Osmium tetroxide and glutaraldehyde and dehydrated in alcohol. The samples were air-dried at room temperature (25 °C). Each sample was coated with a thin layer of conducting material (gold/palladium) using a sputter coater before SEM measurement and examined under a scanning electron microscope (Philips XL30, Netherlands, available at CIRCOT, Mumbai) with an accelerating voltage of 15 or 17 kV.

### Statistical analysis

Each tank (with 15 fish in each) constituted as an experimental replicate for data on growth, while fish per se were units for rest of the data such as biochemical and immunological measurements. All data were analyzed using Statistical Package for the Social Sciences program version 16.0 for windows (SPSS Inc., Chicago, IL, USA) unless otherwise indicated. The data were tested for Gaussian normal distribution (Shapiro–Wilk’s normality test) and homogeneity of variance (Levene’s test). Log or arcsine transformation of data was done prior to statistical analysis whenever necessary. Two-way analysis of variance (ANOVA) was used to test the effect of FOS level and BS level (main effects) along with their interaction. When interaction was significant, one-way ANOVA was used to determine whether any significant variation existed between the treatments. When overall differences were found, the means were tested by Duncan’s multiple range test. Comparison of pre-and post-challenge mean values was done by Student’s t-test. All differences were considered significant at 5% (p < 0.05) and the results are presented as mean ± SEM (standard error of the mean). Kaplan–Meier survivorship curves analysis was done on Graphpad prism 6 software for Windows to estimate the cumulative survival of fish while the log-rank (Mantel–Cox) test for pairwise comparisons were used to detect the significant differences among groups.

## Results

### FOS and *B. subtilis* synbiotic combination promoted growth and performance is not necessarily additive and protease and microvilli attributes supports it

**Figure 1:**
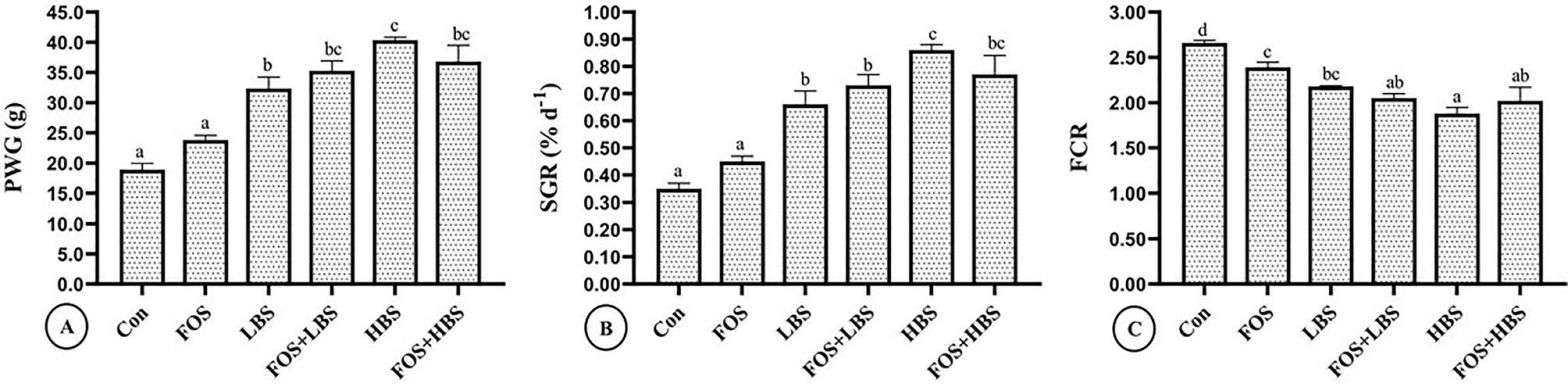
Growth performance of *L. fimbriatus* fingerlings fed diets supplemented with prebiotic, fructooligosaccharide (FOS) and probiotic, *B. subtilis* (BS) alone or in combinations for 60 days. (**A**) PWG—percentage weight gain, (**B**) SGR—specific growth rate, (**C**) FCR—feed conversion ratio. Treatments: Con: Control: without BS/FOS, FOS: 0.5% FOS alone, Low BS (LBS): BS at 1 × 10^4^ alone, FOS + LBS: 0.5% FOS and BS at 1 × 10^4^ CFU, High BS (HBS): BS at 1 × 10^6^ CFU alone, and FOS + HBS: 0.5% FOS and BS at 1 × 10^6^ CFU. Data represented as means ± S.E.M. (*n* = 3). BS and FOS interactions effect are presented in the chart and bars sharing same small alphabet letter are not significantly different (*p* > 0.05). Main effects (FOS and BS) from Two-way ANOVA are presented in the results section. Significance of all tests was accepted at *p* = 0.05.

Two-way ANOVA indicated that FOS did not affect growth (*p* > 0.05) and feed utilisation parameters, such as PWG (*p* = 0.299), SGR (*p* = 0.424), and FCR (*p* = 0.173); however, dietary probiotic bacteria, at 10^4^ or 10^6^ CFU incorporation level, had significant effects (*p* ≤ 0.001) on these parameters, in comparison to the control. Furthermore, fish growth and feed efficiency parameters were similar in groups with 10^4^ or 10^6^ CFU of BS g^−1^ of diet (Fig. 1).

The PWG was 113% and 71% more in fish fed with 10^6^ or 10^4^ CFU of BS g^−1^ of diet, respectively, compared to the control. Similarly, feeding the fish with diets incorporated with 10^4^ or 10^6^ CFU of BS g^−1^ of diet improved SGR by 88.6% and 145%, and FCR by 18% and 29%, respectively. Moreover, there was a significant statistical interaction between FOS and BS on PWG (*p* = 0.015), SGR (*p* = 0.018) and FCR (*p* = 0.035). Compared to FOS alone, BS supplementation at 10^4^ or 10^6^ CFU with FOS significantly improved PWG, SGR and FCR by 55, 71 and 15%, respectively (Fig. 1).

**Figure 2:**
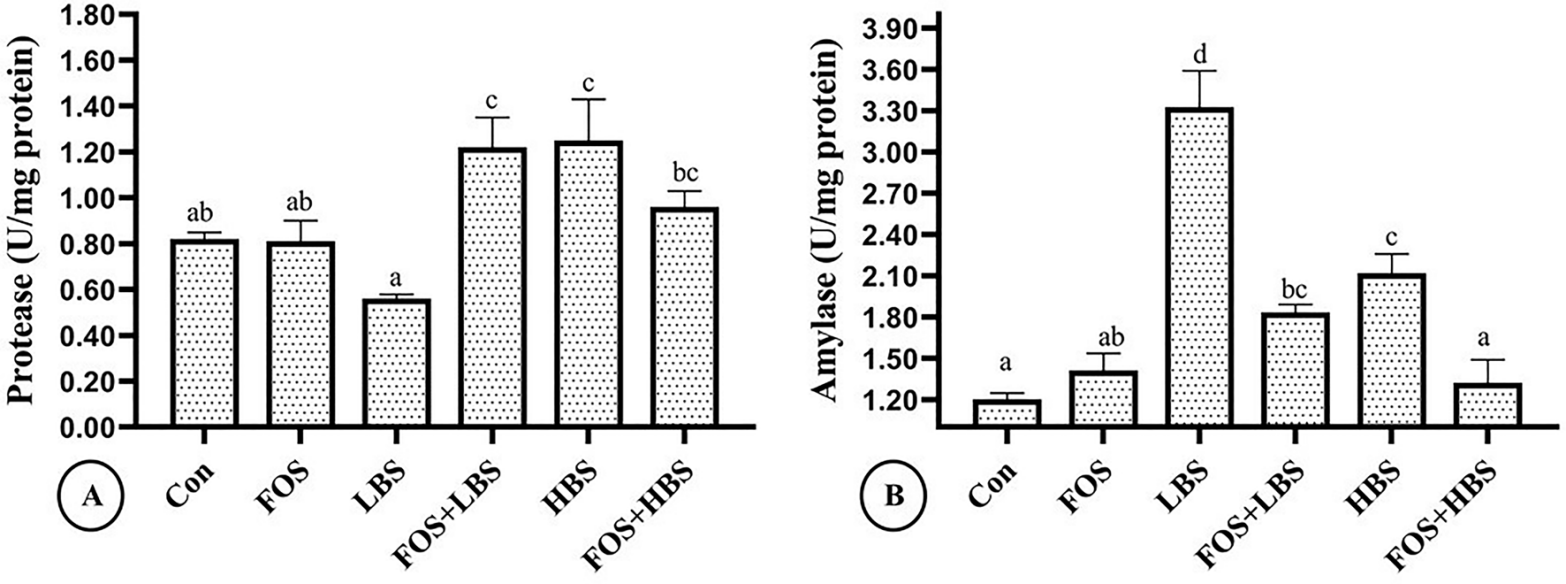
Intestinal protease and amylase activity of *L. fimbriatus* fingerlings fed diets supplemented with fructooligosaccharide (FOS) and *B. subtilis* (BS) alone or in combinations for 60 days. (**A**) Intestinal protease activity, (**B**) Intestinal amylase activity. Treatments: Con: Control: without BS/FOS, FOS: 0.5% FOS alone, Low BS(LBS): BS at 1 × 10^4^ alone, FOS + LBS: 0.5% FOS and BS at 1 × 10^4^ CFU, High BS (HBS): BS at 1 × 10^6^ CFU alone, and FOS + HBS: 0.5% FOS and BS at 1 × 10^6^ CFU. Data represented as means ± S.E.M. (*n* = 3). BS and FOS interactions effect are presented in the chart and bars sharing same small letter alphabet are not significantly different (*p* > 0.05). Main effects (FOS and BS) from Two-way ANOVA are presented in the results section. Significance of all tests was accepted at *p* = 0.05.

There was a statistically significant interaction between FOS and BS on the protease activity (2-way ANOVA, *p* = 0.001; Fig. 2A). Feeding the fish with FOS or LBS, in isolation, had no significant effect on the protease activity. However, when the fish were fed with only HBS supplemented diet, or diet supplemented with LBS and FOS, a significant increase in the protease activity, in comparison to the control and the rest of the treatment groups, was observed (*p* < 0.05). Furthermore, fish fed with a combination of FOS and HBS demonstrated 23% reduced protease activity, in comparison to the HBS group. The protease activity in the fish fed with FOS and HBS was statistically similar to the control and the FOS only group.

The FOS and BS also had a significant interactive effect on the amylase activity (2-way ANOVA, *p* < 0.001; Fig. 2B). Inclusion of only FOS in the diet, did not affect amylase activity. On the contrary, the fish that received only 10^4^ (LBS) or 10^6^ (HBS) CFU of BS g^−1^ of diet demonstrated a 178% and 77% higher amylase activity, in comparison to the control (*p* < 0.001). Furthermore, addition of FOS to the diets containing LBS or HBS, reduced amylase activity by 17 and 38%, in comparison to the LBS or HBS only treatment groups, respectively. Amylase activity in the fish fed with FOS, in combination with LBS, was approximately 53% higher than the control but statistically similar to the FOS only group. The amylase activity in the fish fed with a combination of FOS and HBS was statistically similar to the control and FOS only group.

**Figure 3:**
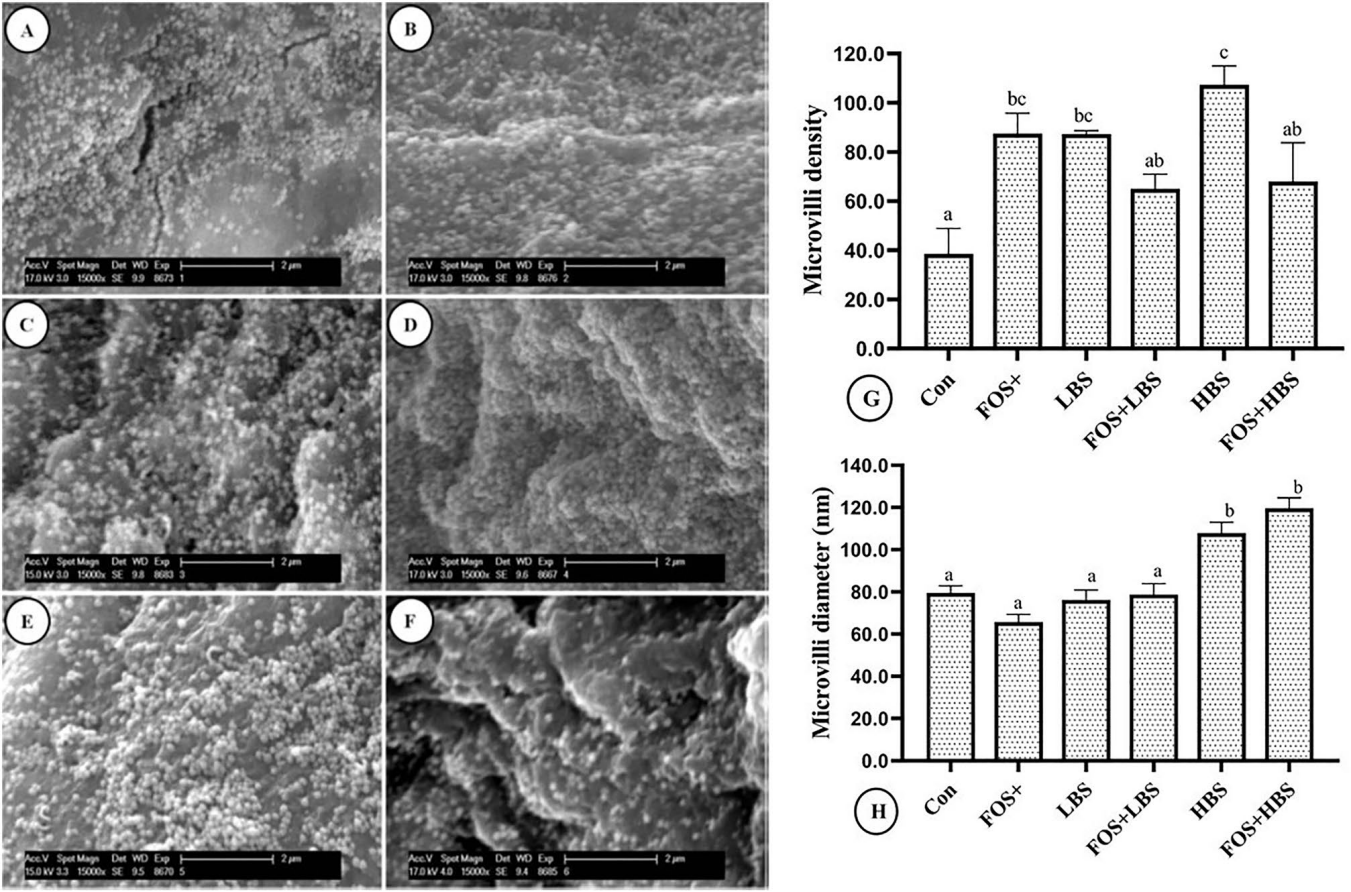
Scanning electron micrographs of intestinal microvilli and their density in *L. fimbriatus* fingerlings fed diets supplemented with fructooligosaccharide (FOS) and *B. subtilis* (BS) alone or in combinations for 60 days. (**A**) FOS (0.5% level), (**B**) LBS (10^4^ CFU/g BS), (**C**) FOS + LBS (0.5% FOS and 10^4^ CFU/g BS), (**D**) HBS (10^6^ CFU/g BS), (**E**) FOS + HBS (0.5% FOS and 10^6^ CFU/g BS) and (**F**) Control (without FOS or BS). (**G**) Microvilli density (average number of microvilli present on the surface of enterocytes standardised to a 4 μm^2^ region; means ± S.E.M: *n* = 4). (**H**) Microvilli diameter (average diameter of microvilli fed with FOS and BS supplemented diets alone or in combination; means ± S.E.M: *n* = 10). BS and FOS interactions effect are presented in the chart and bars sharing same small letter alphabet are not significantly different (*p* > 0.05). Main effects (FOS and BS) from Two-way ANOVA are presented in the results section. Significance of all tests was accepted at *p* = 0.05.

Data and pictures of intestinal scanning electron micrograph of *L. fimbriatus* fingerlings fed dietary FOS and BS are presented in Fig. 3. There existed a statistically significant interaction between FOS and BS on microvilli density and diameter (2-way ANOVA, *p* < 0.02). Feeding fish only with dietary FOS, LBS or HBS increased the intestinal microvilli density by 2 to 4-folds, in comparison to the control (*p* < 0.01; Fig. 3G). Interestingly, when the fish were fed with the diets combining FOS with BS, the microvilli density was statistically similar to that in the fish from control (Fig. 3G).

**Figure 4:**
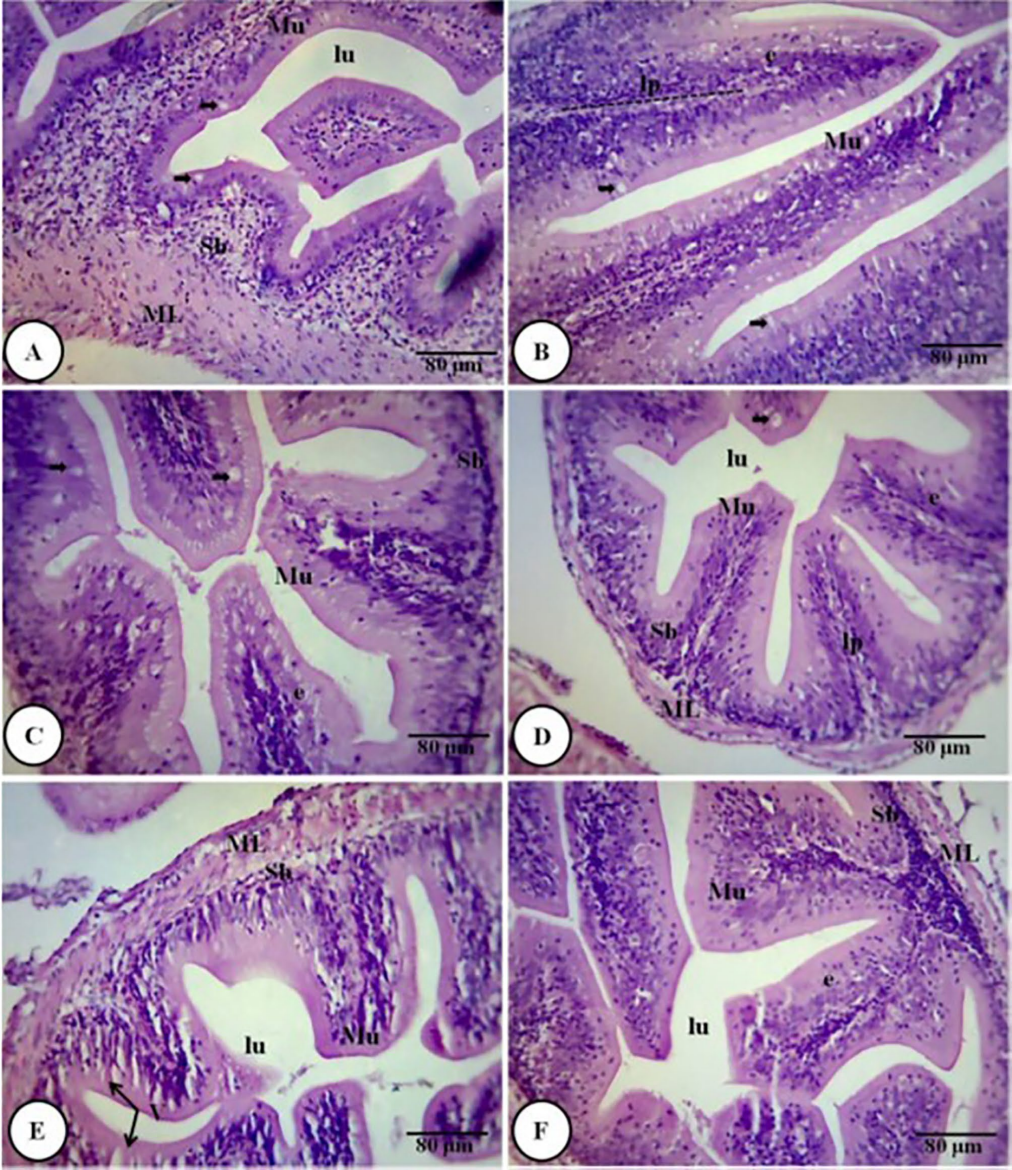
Intestinal histology of gastrointestinal tract of *L. fimbriatus* fingerlings fed diets supplemented with fructooligosaccharide (FOS) and *B. subtilis* (BS) alone or in combinations for 60 days. (**A**) FOS (0.5% level), (**B**) Low BS(LBS) (10^4^ CFU/g BS), (**C**) FOS + LBS, (**D**) High BS (HBS) (10^6^ CFU/g BS), (**E**) FOS + HBS and (**F**) Control (No FOS, NO BS i.e. without BS/FOS). BS and FOS interactions effect are presented in the chart and bars sharing same small letter alphabet are not significantly different (p > 0.05). Main effects (FOS and B. *subtilis*) from Two-way ANOVA are presented in the results section. Significance of all tests was accepted at *p* = 0.05. Lu, lumen; Mu, Mucosa; Sb, Submucosa; ML, Muscularis; V, Absorptive vacuoles; lp, lamina propria; e, epithelial layer; goblet cell (arrowhead).

The intestinal microvilli diameter (Fig. 3H) in the fish fed with HBS, alone or in combination, with FOS, was 25–50% percent more than in the control group. Whereas the microvilli diameter in the intestines of the fish that were not fed with HBS, was statistically similar to each other and to the control. The intestinal histological sections (Fig. 4) showed a uniform mucosal lining in all the dietary treatments, except in the fish fed with FOS + LBS and FOS + HBS, with a significant increase in the number of goblet cells and hyperplasia of these cells in the later (Fig. 4).

### FOS and *B. subtilis* synbiotic combination promoted haemato-serological effects are additive during pre- and or post- challenge

**Figure 5:**
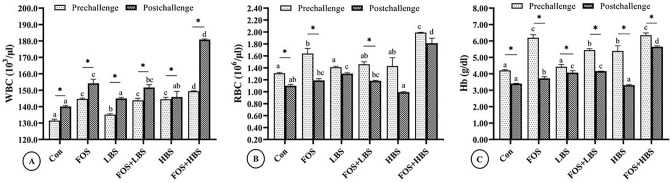
Hematological changes in *L. fimbriatus* fingerlings challenged with pathogenic bacteria, *A. hydrophila* and fed diets supplemented with fructooligosaccharide (FOS) and *B. subtilis* (BS) alone or in combinations for 60 days. (**A**) White blood cells (WBC) count, (**B**) red blood cells (RBC) count, and (**C**) hemoglobin (Hb). Treatments: Con: Control: without BS/FOS, FOS: 0.5% FOS alone, Low BS(LBS): BS at 1 × 10^4^ alone, FOS + LBS: 0.5% FOS and BS at 1 × 10^4^ CFU, High BS (HBS): BS at 1 × 10^6^ CFU alone, and FOS + HBS: 0.5% FOS and BS at 1 × 10^6^ CFU. Data represented as means ± S.E.M. (*n* = 3). BS and FOS interactions effect are presented in the chart and bars sharing same small letter alphabet are not significantly different (p > 0.05). Main effects (FOS and BS) from Two-way ANOVA are presented in the results section. Significance of all tests was accepted at *p* = 0.05. Asterisks symbolize significant difference between (pre-) and (post-) challenge treatment groups (*T*-test; *p* < 0.05).

A 2-way ANOVA identified a statistically significant interactive effect of FOS and BS on the WBC count in the fish before and after the challenge with pathogenic *A. hydrophila* (*p* < 0.001). Injecting fish with the pathogenic bacteria increased the leucocyte count in all the fish after 10 days (Fig. 5A). Before being challenged with the pathogenic bacteria, the inclusion of FOS or BS, alone or in combination, increased the total leucocyte count in comparison to the control (Fig. 5A). The highest WBC count, among all the treatment groups, was found in fish fed with FOS and HBS, followed by that in the fish fed with FOS, HBS, or FOS + LBS supplemented diet. The WBC count was least among all the treatment groups, but control, in the fish fed with 10^4^ CFU of BS g^−1^ of diet. Similar to the pre-challenged fish, the WBC count in the fish post-challenge was highest in the fish fed with FOS + HBS, in comparison to the control (*p* < 0.001). The WBC count was also higher than the control in the fish fed with FOS or FOS + LBS post challenge (*p* < 0.05). However, the WBC count in the fish fed only with LBS or HBS was statistically similar to the post-challenge levels found in the control.

A statistical interaction between FOS and BS was also found on the RBC count of both pre- and post-challenged fish (2-way ANOVA, *p* < 0.01). Challenge with pathogenic bacteria lowered the numeric count of RBC in all the fish, with statistically significant effects observed in control and fish fed with FOS, alone or in combination with LBS (*p* < 0.001; Fig. 5B). In the pre-challenged fish, the RBC count was found significantly higher than the control in the fish fed with FOS alone or FOS with HBS (*p* < 0.01), with highest count in the latter. However, post-challenge with the pathogenic bacteria, the RBC count was statistically higher in the fish fed with 10^4^ CFU of *B. subtilis* g^−1^ of diet and FOS + HBS (Fig. 5B).

There was a statistically significant interaction between FOS and BS on the Hb levels of the fish, before and after the challenge with *A. hydrophila* (*p* = 0.026 and *p* < 0.001, respectively). In the pre-challenged fish, the Hb concentration significantly increased in all the dietary treatments, except when fed with LBS, when compared to the control group (*p* < 0.05; Fig. 5C). The Hb concentration in the pre-challenged fish fed with FOS or FOS + HBS was approximately 50% higher than the control, whereas it was 28–30% greater in the fish fed with diets supplemented with HBS or FOS + LBS. As a general trend, the Hb concentration in all the post-challenge fish was significantly lower than the pre-challenge levels. The Hb concentration amongst all the post-challenged fish was elevated, when compared to the post-challenged control fish, except when fed with diets containing only BS at 10^6^ CFU g^−1^ of the diet without FOS. The Hb concentration was highest in fish fed with FOS + HBS, followed by the groups that received LBS or LBS + FOS supplemented diets. Supplementing diet only with FOS also elevated Hb levels marginally but in a statistically significant manner. Further, blood glucose concentration in all the pre-challenge treatment groups was significantly lower than control group (Fig. S2: Supplementary).

**Figure 6:**
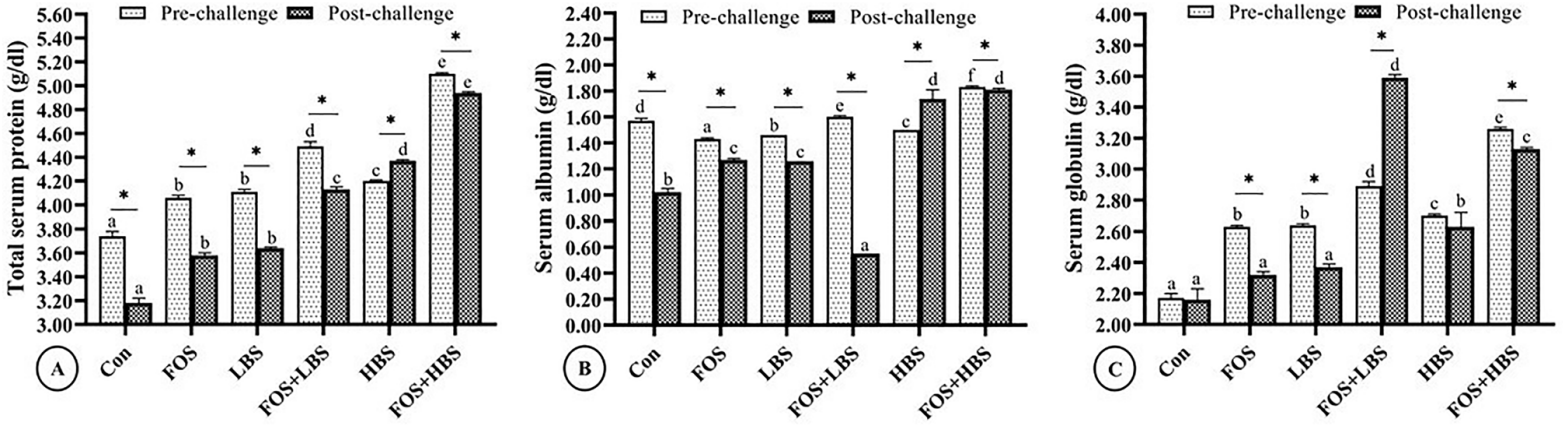
Serum protein profile of *L. fimbriatus* fingerlings challenged with pathogenic bacteria, *A. hydrophila* and fed diets supplemented with fructooligosaccharide (FOS) and *B. subtilis* (BS) alone or in combinations for 60 days. Bars are indicative of means of values parameter prior to (pre-) and (post-) challenge. Treatments: Con: Control: without BS/FOS, FOS: 0.5% FOS alone, Low BS(LBS): BS at 1 × 10^4^ alone, FOS + LBS: 0.5% FOS and BS at 1 × 10^4^ CFU, High BS (HBS): BS at 1 × 10^6^ CFU alone, and FOS + HBS: 0.5% FOS and BS at 1 × 10^6^ CFU. Data represented as means ± S.E.M. (*n* = 3). BS and FOS interactions effect are presented in the chart and bars sharing same small letter alphabet are not significantly different (p > 0.05). Main effects (FOS and BS) from Two-way ANOVA are presented in the results section. Significance of all tests was accepted at *p* = 0.05. Asterisks symbolize significant difference between (pre-) and (post-) challenge treatment groups (*T*-test; *p* < 0.05).

The effect of the dietary treatments on total protein and globulin is illustrated in Fig. 6. There was a significant interactive effect of FOS and BS on the serum protein and globulin levels of the fish, before and after the challenge study (2-way ANOVA; *p* < 0.001). The serum total protein content was statistically higher in both pre-challenge and post-challenge fish fed with diets containing BS, alone or in combination with FOS. Significantly synergistic effects of FOS and HBS were noticed on total serum proteins, and the values exceeded all other groups. The serum protein in pre- and post-challenge fish fed with FOS + HBS was 36% and 55% higher than the control, respectively. Similarly, the protein content in fish fed with HBS alone, in the pre- and post-challenge fish, was 12.3% and 37% more than corresponding controls, respectively. The serum total protein content in all the post-challenge fish was statistically lower than their pre-challenge levels, except the HBS supplemented fed fish where the values significantly increased in the post-challenge group compared to its respective pre-challenge group. In the pre-challenged fish, serum albumin content was lower than the control due to dietary inclusion of FOS, LBS, or HBS alone (*p* < 0.05); however, dietary combination of prebiotic (FOS) with either HBS or LBS increased the serum albumin concentration in comparison to the control. In the post-challenged fish, the serum albumin concentration was lower than the control when fish were fed with FOS + LBS. On the contrary, the serum albumin concentration was high in all the other treatments when compared to the control. The serum albumin concentration in the post-challenge fish was 71% and 77% higher when fish were fed with HBS, alone or with FOS, respectively. The values were around 25% higher in FOS and LBS alone.

The serum globulin concentration was 33% and 50% higher than the control in the pre-challenged fish fed with FOS, in combination with LBS or HBS, respectively (Fig. 6C). Feeding HBS only also caused a modest increase (~ 24%) in the serum globulin concentration in comparison to the control. The globulin concentration in the control did not change between the pre-challenged and post-challenged fish. Similarly, the bacterial challenge had no significant effect on the fish that received diet with HBS. However, the serum globulin level in the fish fed with a combinatorial mix of FOS and LBS was 24% higher in the post-challenged fish, in comparison to the pre-challenged condition. The average serum globulin concentration in the post-challenged fish was low, as compared to the pre-challenge levels, in the rest of the treatment groups. The overall trend of the change in the serum globulin concentration of the post-challenged fish was similar to that observed in the pre-challenged condition.

### FOS and *B. subtilis* synbiotic combinations’ additive immunological effects during pre- and or post- challenge explains increased survival after pathogen infection

**Figure 7:**
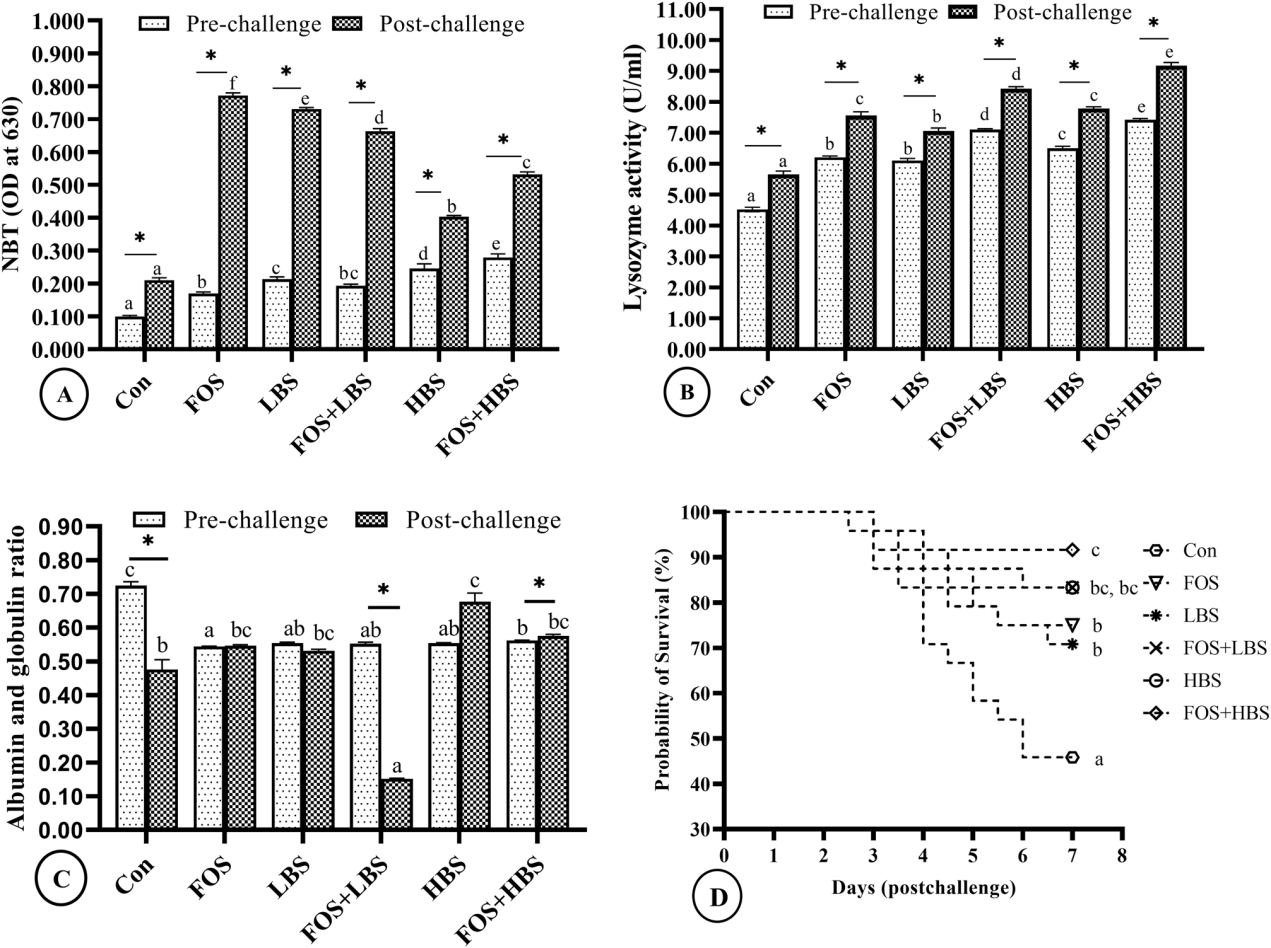
Immunological responses and Kaplan–Meier survivorship curve of *L. fimbriatus* fingerlings fed diets supplemented with fructooligosaccharide (FOS) and *B. subtilis* (BS) alone or in combinations for 60 days. (**A**) NBT: Nitroblue tetrazolium test, (**B**) lysozyme activity, (**C**) albumin globulin ratio, and (**D**) Kaplan–Meier survivorship cure depict probability of survival (%) over time (days). Treatments: Con: Control: without BS/FOS, FOS: 0.5% FOS alone, Low BS(LBS): BS at 1 × 10^4^ alone, FOS + LBS: 0.5% FOS and BS at 1 × 10^4^ CFU, High BS (HBS): BS at 1 × 10^6^ CFU alone, and FOS + HBS: 0.5% FOS and BS at 1 × 10^6^ CFU. Data represented as means ± S.E.M. (*n* = 3). BS and FOS interactions effect are presented in the chart and bars sharing same small letter alphabet are not significantly different (*p* > 0.05). Main effects (FOS and BS) from Two-way ANOVA are presented in the results section. Significance of all tests was accepted at *p* = 0.05. Asterisks symbolize significant difference between (pre-) and (post-) challenge treatment groups (*T*-test; *p* < 0.05).

A statistically significant effect of FOS and BS, and their interaction, was found on immunological parameters like NBT, lysozyme activity and A/G ratio (*p* < 0.006). Moreover, post-challenge survival was also significantly affected by FOS, BS, and their interaction (*p* < 0.01) (Fig. 7). In the pre-challenged fish, the A/G ratio was low in all the treatments, in comparison to the control, with lowest values in the fish fed with FOS supplemented diet (Fig. 7C). Bacterial challenge significantly reduced A/G ratio in control (34%) and in the fish fed with FOS + LBS (73%), in comparison to the levels observed pre-challenge. In contrast, the A/G ratio was 4% higher in the post-challenge fish fed FOS + HBS in comparison to the pre-challenge levels**.** The A/G ratio, between the pre- and the post-challenged fish, did not change in fish fed FOS only or LBS only (*p* > 0.05). Amongst the post-challenged fish, the A/G ratio was lowest in the fish fed with FOS + LBS (69% lower than the control). In contrast, the A/G ratio was 42% and 21% higher than the control in the fish given HBS, either alone or in combination with FOS, respectively.

The NBT assay, a measure of phagocytotic activity of the immune system, was affected significantly by FOS, BS, and their interaction (2-WAY ANOVA; *p* < 0.001; Fig. 7A). Prior to the challenge with pathogenic bacteria, the NBT value was recorded highest in the serum from the fish that received FOS + HBS (178% above control), followed by the fish fed with only HBS supplemented diet (167% above control). In the fish fed diet with LBS, alone or with FOS, the NBT values were similar but 111% and 113% higher than the control, respectively. The NBT value from the fish given FOS supplemented diet was 89% above that in the control. The NBT values were higher in the post-challenged fish, in comparison to the values observed in the pre-challenged fish of the corresponding treatments. For example, the NBT values of the control fish, after bacterial challenge, increased by 133% above the values observed in the pre-challenged control fish. Amongst the post-challenged fish, the NBT values in fish fed diet supplemented with FOS or LBS alone increased by 267% and 248% than that observed in its control. The NBT values in the post-challenged fish that were fed with FOS + LBS, HBS or FOS + HBS was 214%, 90%, and 152% higher than the control, respectively.

Dietary inclusion of probiotic (BS) or prebiotic (FOS) had significant effect on the serum lysozyme activity of both pre- and post-challenged fish. A statistically significant interactive effect between BS and FOS on the lysozyme activity was also present (*p* < 0.001). In pre-challenged state, the lysozyme activity increased in all the treatments, with 64% and 57% more activity in the fish fed FOS with HBS or LBS, in comparison with the control. The lysozyme activity due to HBS alone was about 44% more than the control. The lysozyme activity due to only FOS or LBS inclusion was similar to each other, but about 37% more than what was observed in the serum from the fish in control (Fig. 7B). The lysozyme activity due to exposure to pathogenic bacteria was increased in all the fish, including the control, compared to the corresponding pre-challenge group. In the post-challenged fish too, the lysozyme was elevated in the serum due to dietary FOS, LBS and HBS alone, or in combinations with further significant dose dependent potentiation.

The survival of the fish, post-challenge, was also significantly affected by dietary treatments and the interaction between FOS and BS. The survival in the control group was only 46%, whereas survival in the fish fed with FOS, LBS, HBS, or FOS + LBS was 75, 71, 83 and 83%, respectively. The highest survival (92%) was observed in the group of fish fed with FOS + HBS (Fig. 7D).

The cumulative survival (%)/mortality in disease exposed fish was recorded between day 2 and 7 post-challenge. At the end of the challenge period, neither signs of infection nor mortalities were observed in fish inoculated with sterile solution (control − ve), whereas varying levels of mortality were observed in fish exposed to *A. hydrophila* O:18 depending on the experimental diet (Table S2: Supplementary). Fish infected with pathogenic bacteria *Aeromonas hydrophila* exhibited one or more typical signs of infection (Fig. S3: Supplementary). The reported common gross signs of disease included large and irregular haemorrhages, shallow to deep necrotizing ulcers, and abdominal distension with sero-hemorrhagic fluids drained from the inflamed vent.

## Discussion

Our results demonstrate that prebiotic (FOS), on its own, had no significant effect on the growth or feed utilization in *L. fimbriatus*. In contrast, all the treatments that received BS, alone or in combination with FOS, showed an increased growth and better feed utilization (Fig. 1A–C). The results infer that FOS, at 5% inclusion level, does not improve fish growth performance in *L. fimbriatus*. Our results are also substantiated by previous studies. For example, incorporation of FOS at 0.5% dose had no effect on growth of tilapia^71^. Similarly, FOS at 1% had no significant effect on growth of Atlantic salmon (*Salmo salar*)^72^ or white sea bream (*Diplodus sargus*)^73^. However, it should be noted that few studies have also demonstrated growth promoting effects due to FOS in fin and shellfish which could be attributed to varying dosage of FOS and dietary composition or gut flora^71,74^. Though FOS on its own may not affect fish growth, it can provide suitable substrate for probiotic bacteria to establish and thereby promote growth and feed efficiency in fish^12,71^. The interactions between FOS and BS observed in our study in promoting fish growth and dietary efficiency, provides evidence that synbiotic combination of pre and probiotics provide better growth performance, in comparison to their individual applications^12,36,37^. These results are in conformation with those reported earlier^40^, wherein three biotic combinations (FOS + *B. clausii*, MOS + *B. clausii* and FOS + MOS + *B. clausii*) in Japanese flounder (*Paralichthys olivaceus*) yielded better growth compared with their individual application.

The growth enhancing properties of dietary probiotics are linked to their ability to secrete extracellular enzymes (protease, amylase, cellulose, phytase, chitinase, lipase etc.) and vitamins to support digestive function^75–77^. A large number of previous studies have already reported beneficial effects of probiotic bacteria on several carp species, including *L. rohita*^11,22,30,78^. However, *B. subtilis* and most other probiotic bacteria require a suitable fermentation substrate to establish their colonies^79^. Our results indicate that FOS could act as a substrate (prebiotic) for the bacteria to grow and exert their positive effects. Although, our observations reveal an interactive effect between FOS and BS, a synbiotic relationship, their biological effect was non additive in nature. Hence, a FOS and BS synergism may not always show a linear beneficial relationship in response to the varying dose of FOS or BS. A similar non-linear growth performance, in response to the dose of BS or FOS was also observed in juvenile large yellow croaker, *Larimichthys crocea*^38^, sea cucumber, *Apostichopus japonicus*^80,81^ and ovate pompano, *Trachinotus ovatus*^82^. Contrary to this, synergistic actions between mannan oligosaccharides and *Bacillus* spp., mannan oligosaccharides and *Enterococcus faecalis*, MOS/FOS and *Bacillus* spp., FOS and *Bacillus* spp. was revealed in studies on rainbow trout, *Oncorhynchus mykiss*^36^, European lobster larvae, *Homarus gammarus L*.^43^, Triangular bream, *Megalobrama terminalis*^83^ and Japanese eel, *Anguilla japonica*^33^. Our previous work with *L. fimbriatus* fingerlings showed a significantly higher growth performance when fed with a 0.5% MOS and 10^4^ CFU of *B. subtilis* g^−1^ supplemented diet^22^ and synergistic effect was evident between MOS and BS. Biological synergism is a complex phenomenon affected not only by the prebiotic and probiotic relationship, but also by their dosage, species of bacteria and selection of prebiotic. Further, the type of fish species and rearing environment are also important as they will allow a conducive atmosphere for the bacterial colonies to establish in the gastrointenstinal tract^10,84,85^.

Digestive enzymes play a significant role in digestive function of fishes and their estimation gives a reliable information on the ability of fish to hydrolyse feed constituents like carbohydrates, proteins, and lipid^84^. In addition to the innate enzymes, the gut microbiota also alters or aid digestibility of feed, along with the influence on disease resistance, immunity and energy homeostasis in fish^86–88^. In the present study, feeding of 10^6^ CFU of *B. subtilis* g^−1^ of diet, significantly enhanced protease activity in the fish. A similar increase in the activity of digestive enzymes was reported in *M. rosenbergii* (Giant freshwater prawn) post-larvae with *B. subtilis* (MTCC-121)-supplemented diets^21^. *Bacillus subtilis* LS 1–2 was also shown to improve broiler intestinal health and microbial balance^89^. High protease activity due to FOS + LBS, in comparison with LBS or FOS alone clearly alludes to a synergistic interaction, termed as synbiotic relationship^90^. However, a similar synergism was not observed with FOS + HBS, thus, suggesting a non-additive probiotic-prebiotic relationship. This possibly relates to the inherent capacity of *Bacillus sp*. to produce a wide range of vitamins (e.g. Vitamin K and B-12) and extracellular enzymes (e.g. esterase, protease and amylase) at specified inclusion level^85,91^. Adorian et al.^92^ observed higher digestive enzyme activity in *Lates calcarifer* fed diets supplemented with *B. licheniformis* and *B. subtilis* at 1 × 10^6^ CFU g^−1^ than at 1 × 10^9^ CFU g^−1^ feed^92^. Triangular bream, *M. terminalis* fed with FOS and *B. licheniformis* alone or in combination were reported to improve the intestinal digestive activities^83^. Further, dietary supplementation of probiotic, *Saccharomyces cerevisiae*, and prebiotic, galactooligosaccharide (GOS), at 2.5 g kg^−1^ had significantly improved the growth and digestive enzyme activities in *Channa punctatus*^93^. The amylase activity also did not show a linear trend in response to the addition of prebiotic or probiotic in the diet. The amylase activity was more than twice in the fish fed with LBS, in comparison to that observed in the fish given HBS supplemented diet (Fig. 2B). Moreover, addition of FOS with LBS or HBS reduced the amylase activity in comparison to what was observed in the fish given only LBS or HBS-containing diets, respectively. Therefore, the higher inclusion levels of BS alone or in combination with FOS was counterproductive for the amylase activities in *L. fimbriatus* fingerlings. Previously, studies have indicated that the amylase activity in the fish gut is sensitive to the amount of inclusion of prebiotic in the diet and may not always respond positively to the higher inclusion level. For example, the intestinal amylase activity in blunt snout bream (*M. amblycephala*)^94^ fingerlings and crucian carp (*C. auratus gibelio*)^95^ decreased when prebiotic inclusion level increased beyond 4 and 2.1 g kg^−1^ of diet, respectively.

The regulation of glucose metabolism and the production of associated digestive enzymes in fish fed dietary pre- and pro-biotics is poorly understood. Salivary amylase activity in humans is known to enhance glucose homeostasis^96^. Similarly, pancreatic amylase activity was also shown to participate in the maintenance of postprandial glucose homeostasis in pig model^97^. The main source of amylase in fish is the cells of the exocrine pancreas and gut microbiota^98^. In the present study, the exogenous supplementation of pre-and probiotics additives in diet of *L. fimbriatus* enhanced amylase activity as well as improved glucose homeostasis among the different dietary treatments when compared with control group. There are currently very limited studies on digestibility of carbohydrates in probiotic supplementation in fish and available reports related to in vitro observations only^99^. Our previous work with MOS and BS in *L. fimbriatus* fingerlings showed a significantly higher intestinal protease activity with 0.5% MOS and 10^6^ CFU of BS g^−1^ of diet, in comparison to 0.5% MOS alone; however, the intestinal amylase activity was also higher with MOS and BS combination but did not differ, with respect to the control and with MOS alone^22^. It is apparent that the discrepancies in the findings are based on the inclusion levels of additives, chemical composition of substrate and the probiont strain used. Moreover, synergistic effect is based on a designed synbiotic combination wherein provided substrate is utilized by co-administered probiont that provides a competitive advantage to a probiont over competing endogenous populations, thus effectively improving the survival and implantation of the live microbial dietary supplement in the gastrointestinal tract of the host^17,34^.

Dietary prebiotics can modulate intestinal microbial communities and change intestinal histomorphology, including gut absorptive surface area, length and density of microvilli, epithelial brush border in European sea bass, seabream, rainbow trout, salmon and larval cobia^28,100–104^. In fact, it has been reported that the synbiotic combination of *B. subtilis*, *B. licheniformis* and isomaltooligosaccharides can enhance the growth and activity of gut microbial populations in *P. japonicus*^42^ and *Salmo trutta caspius*^105^. Consequent to the alterations in the gut health and morphology of the host, increased growth and better feed efficiency is observed^15^.Feeding fish with HBS, alone or in combination with FOS, caused an increase in both microvilli diameter and density which is an indicator of larger absorptive surface for better absorption and assimilation of gut nutrients^100,103^. Thus, the observed higher microvilli density and altered gut enzymatic activity could be attributed to overall growth performance and improved feed efficiency of *L. fimbriatus* when compared with control. The possibility of improved gut health and morphology can be attributed to a specific composition of synbiotic that might have stimulated better colonisation of the given probiont (synergistic synbiotic) or selectively stimulated endogenous microbiota (complementary synbiotic) for improved utilisation of dietary carbohydrate and fatty acids^106,107^. Therefore, it is essential to identify optimal inclusion level of probiotic, prebiotic, and their relative concentrations for positive results. We could not assess the implications of our dietary treatments on the gut microbial biodiversity. Further studies can ascertain how the synbiotic mixture of FOS and BS affect gut microflora to obtain a better picture of gut health.

Another important histological observation in fish fed combined FOS + LBS and FOS + HBS was increased number of goblet cells along with hyperplasia of these cells in the later (Fig. 4). Reports on probiotic feeding indicate both increase^108–111^ and decrease^112^ in number of goblets. The significant variations or no changes in number of these cells appears to depend on the species, gut part examined (proximal, middle or distal), dose, type, combinations of prebiotic and probiotic and their method and length of delivery. For example, in Nile tilapia, mixed bacteria probiotic (PRO) + MOS, PRO + chitosan and PRO feeding for 63 days have been found to stimulate an increase in goblet cells^113^. On contrary, in European sea bass (*Dicentrarchus labrax*) juveniles, goblet cells density was not affected by feeding MOS, *Pediococcus acidilactici* or its combination for 90 days^114^. Studies in common carp (*Cyprinus carpio*)^115^ indicates that feeding duration of 90 days or more is essential for effectuation of effects of probiotic combination (L. plantarum + L. rhamnosus) to significantly increase goblet cells and other histomorphological attributes. In yet another 98 days (14-week) feeding trial in *O. niloticus*^111^, though both β-glucan and *Bacillus coagulans* individually were effective to increase goblet cells and the probiotic’s effect was significantly profound than the prebiotic, the effect of synbiotic combinations far exceeded individual impact of these supplements. Goblet cells primarily secrete mucus, which trap and remove/prevent gut pathogens from making epithelial attachments, as well as antimicrobial proteins, chemokines, and cytokines that together activate the local immunity and play a key role in protecting the intestinal barriers^116^. They also passage and deliver soluble antigens of the intestinal lumen to the dendritic /antigen presenting cells in the lamina propria inducing adaptive immune responses^117^. The intrinsic sensing of the gut microbiota by goblet cells is suggested to play a vital role in controlling the immune system’s exposure to challenges by gut microbes^116,118^. Thus, increased abundance of goblet cells in LBS + FOS and HBS + FOS indicates increased localized immune status of the intestine.

Understanding of the haematological scores is considered as an important tool that can be used as an effective index to determine the physiological status and relate the well-being of an aquatic animal^119^. The present study showed an improvement in individual hematological score in both pre and or post-challenged fish. Overall effects of FOS and BS improved RBC, Hb, and WBC count, especially in the fish fed with FOS + HBS. Improvements of haematological scores underline the possible immune-stimulant potency of the supplied probiotic and prebiotics. When compared with control, all treatment groups reported reduced blood glucose before challenge with a pathogenic *A. hydrophila*, suggesting better glucose metabolism and reduced stress^120,121^. In addition, increase in pre- and post-challenge respiratory burst activity was also observed, which confirms the immune stimulating role of these additives. The earlier findings on improved haematological scores in Nile tilapia, *O. niloticus* fed *Micrococcus luteus* and *Pseudomonas* species^122^ and *Huso huso* fed with 0, 1, 2 and 3% dietary oligo-fructose^123^ are in agreement with our present results. Similarly, varied supplementation levels (0, 1, 2 and 4 g/kg diet) of commercial synbiotic in the diet of Beluga, *Huso huso* had significant effect on WBC and Hb counts^124^.

Serum biochemical scores also indicate the overall wellbeing of an animal. In this study, serum scores such as serum total protein, total albumin and total globulin differed significantly among the treatment groups. The interaction effects between FOS and BS level on these parameters during pre- and post-challenge were found to be additive. Remarkable improvement in all treated groups confirms that dietary FOS and BS (alone or combination) supplementation had a positive effect on immune modulation of *L. fimbriatus*. The combined effects of FOS, HBS or LBS on total serum protein and globulin level was more than their individual effect, suggesting an improvement in the non-specific humoral immune function^125^.

Pre- and post-challenge immunological parameters, like respiratory burst activity of leukocytes (NBT), lysozyme activity, albumin: globulin ratio and post-challenge survival were significantly improved by both FOS, BS and their interaction, with maximal effect observed in fish given HBS + FOS supplemented diet (Fig. 7). There was generalised increase in NBT and lysozyme score post-challenge across main effects and interaction. Since lysozyme is also an important humoral non-specific defence protein found in fish mucus, serum and tissues rich in leukocytes^126^, the results further corroborate the findings on positive dose-dependent effects of dietary treatments on immune system. Further, a decreased A/G ratio in all the dietary treatment groups, especially in the FOS + LBS fed post-challenge fish, indicate improved non-specific immunity due to increased globulin level, in comparison to the albumin^127^. With regard to the different combinations used in this study, using 0.5% FOS + 10^6^ CFU/g *B. subtilis* provided significant and highest scores, implying a maximum immune response at this level. Aftabgard et al.^105^ also reported an improvement in gut serum and lysozyme activity from a combined administration of 2 g kg^−1^ isomalto-oligosaccharides and 1 g kg^−1^ BetaPlus^®^ in the basal diet of Caspian brown trout, *S. trutta caspius* fingerlings. Our findings are also in line with our previous work^22,80^. Our study reveals a prebiotic-probiotic synergism, which also conforms to earlier studies in Japanese flounder, *P. olivaceus*^40^ and Sea cucumber, *A. japonicus*^80,81,128^.

Fish experimentally infected with *A. hydrophila* showed characteristic clinical signs like abdominal distension with sero-hemorrhagic fluid oozing from the inflamed vent. Most of the fish displayed irregular haemorrhages, shallow to deep necrotizing ulcers, consistent with the finding of Kumar et al.^129^. However, the effects of improved immune function from probiotic-prebiotic synergism was evident from the increase in survival of *L. fimbriatus* after pathogen challenge. Prior pathological investigations suggest that probiotics maintain a close relationship with the mucosa of fish and induce mucosal immunity^130^, adaptive immune function (B cell and T cell responses) and the complement system^125,131^, in response to the invasion of a pathogen. A similar immune response to *A. hydrophila* infection is evident in the present study. Protective role of FOS in this study may be attributed towards its immune enhancing capacity by providing a useful substrate for beneficial gut micro-biota, which plays a major role in improvement of the immunity^132^. Such synergistic function in *C. mrigala* juveniles fed synbiotic combination of *Bacillus subtilis* + MOS against *A. hydrophila* is well reported by Kumar et al.^133^. Earlier, protective action of *B. subtilis* in *L. rohita*, sea cucumber (*A. japonicus*), large yellow croaker (*L. crocea*) and other fish species against *A. hydrophila* is reported by multiple studies^33,38,80,128,132,134,135^. Previous observations also suggest that synergism between probiotic and prebiotics is dependent on appropriate doses, type of probiotic strain used, type of fish species, feeding habits and rearing environment, etc.^10,36–38^. Thus, it is essential to use the probiotic bacteria at a right dose, in combination with a suitable prebiotic substrate. Non-additive effects of the dietary treatments on the activity of gut enzymes also highlight the importance of optimising the correct probiotic-prebiotic combination and dose.

## Conclusion

Overall findings from the present study affirm the potentiality of the supplemented FOS and *B. subtilis* on growth performance, haemato-serological parameters, immunological attributes and survival after pathogen infection in *L. fimabriatus* fingerlings. The promoted growth performance by FOS and *B. subtilis* synbiotic combination was not additive. However, registered haemato-serological and immunological effects were additive during pre- and or post- challenge that ensured the survivability of fish after pathogen infection. With regards to providing immunity against the pathogenic bacteria, 0.5% FOS + 10^6^ CFU of *B. subtilis* g^−1^ diet was found optimal for *L. fimbriatus.*

## Supplementary Information

Supplementary Information.

## Data Availability

All data supporting the findings are included in this article. The datasets and figures supporting this article have been uploaded as part of the supplementary material.
