# Supplementary material for: Fructooligosaccharide and Bacillus subtilis synbiotic combination promoted disease resistance, but not growth performance, is additive in fish
Source: Sci Rep. 2023 Jul 13;13:11345. doi: 10.1038/s41598-023-38267-7 (PMC10345097; doi:10.1038/s41598-023-38267-7)

**Pawar et al. Supplementary Information**

**Table S1**. Quality parameter of the water used for rearing the *L. rohita* fingerlings.

| Dissolved Oxygen | 6.8 to7.3 mg.L^-1^ |
| --- | --- |
| Temperature | 27–28 °C |
| pH: | 7.2 to 7.9 |
| Free Carbon Dioxide: | Negligible |
| Total hardness | 70-90 mg L^-1^ |
| Ammonia-N | 0.08-0.012 mg L^‑1^ |
| Nitrite-N | 0.001 to 0.002mg L^‑1^ |
| Nitrate-N | 0.04 to 0.06 mg L^‑1^ |

**Table S2:** Challenge studies depicting mortality and relative level of protection percentage.

| Treatment groups | Composition | Subgroups | No. Of challenged fish | No. Of dead fish | Survival (%) | Relative level of protection (%) |
| --- | --- | --- | --- | --- | --- | --- |
| Con | without BS/FOS | C^-ve^ | n=6 | 0 | 0 |  |
|  |  | AH | n=24 | 13 | 45.83 | 0 |
| FOS | 0.5% FOS | C^-ve^ | n=6 | 0 | 0 |  |
|  |  | AH | n=24 | 06 | 75.00 | 53.85 |
| LBS | BS at 1x10^4^ | C^-ve^ | n=6 | 0 | 0 |  |
|  |  | AH | n=24 | 07 | 70.00 | 46.15 |
| FOS+LBS |  | C^-ve^ | n=6 | 0 | 0 |  |
|  |  | AH | n=24 | 04 | 83.33 | 69.23 |
| HBS | BS at 1x10^6^ | C^-ve^ | n=6 | 0 | 0 |  |
|  |  | AH | n=24 | 04 | 83.33 | 69.23 |
| FOS+HBS. |  | C^-ve^ | n=6 | 0 | 0 |  |
|  |  | AH | n=24 | 02 | 91.67 | 84.62 |

Treatments: Con: Control: without BS/FOS, FOS: 0.5% FOS alone, Low BS (LBS): BS at 1x10^4^ alone, FOS+LBS, High BS (HBS): BS at 1x10^6^ and FOS+HBS.

C^-ve^, control negative subgroups injected with sterile phosphate buffer saline solution (PBS).

AH, infected subgroups with pathogenic strain of *A. hydrophila* O:18 at concentration of

1.0 × 10^6^ CFU/mL

**Figure S1**. Fructooligosaccharide sample employed in the experiment.


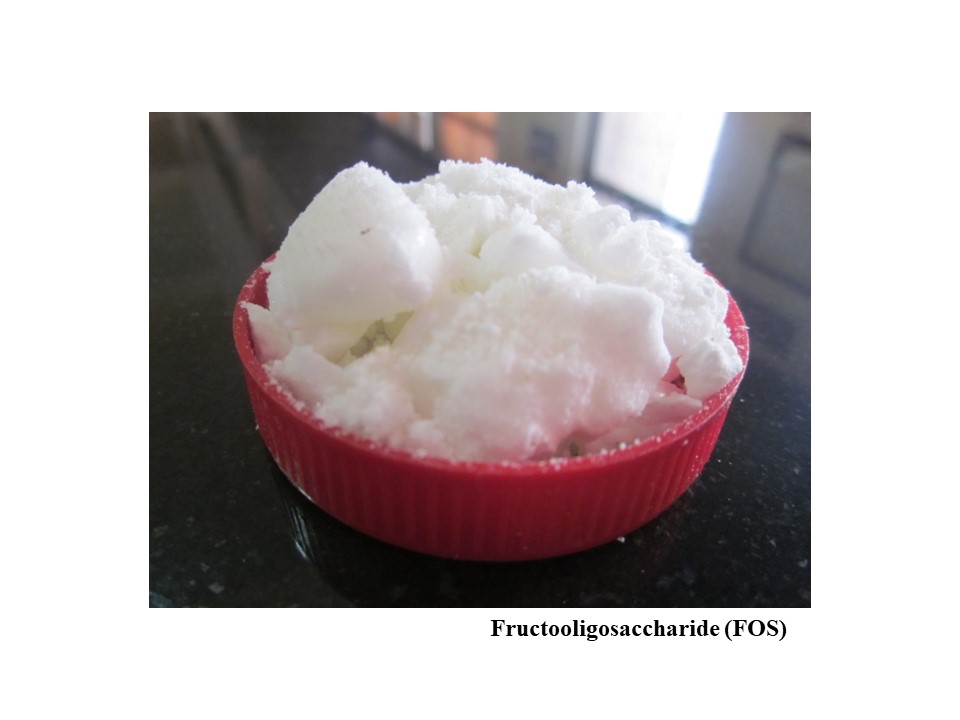


Figure S2. Blood glucose level of *L. fimbriatus* fish fingerlings fed diets supplemented with fructooligosaccharide (FOS) and *B. subtilis* (BS) alone or in combinations for 60 days.


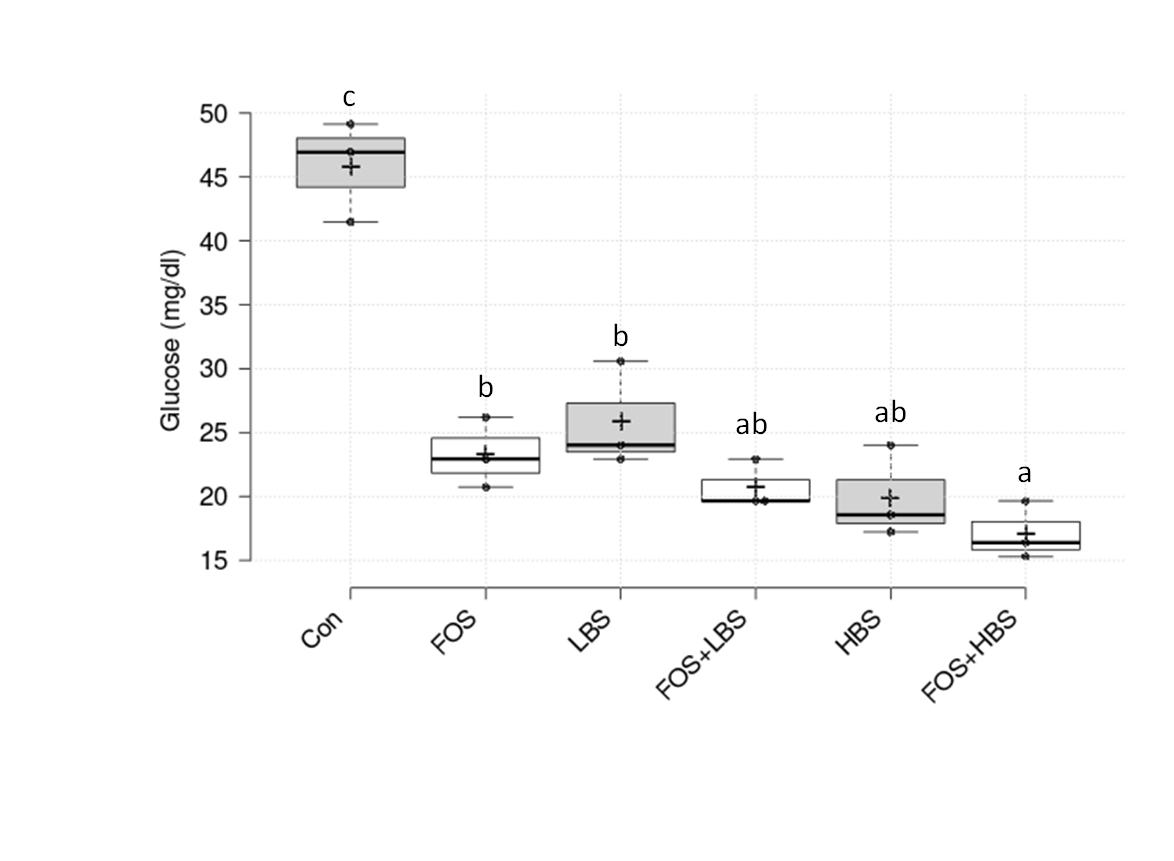


Treatments: Con: Control: without BS/FOS, FOS+: 0.5% FOS alone, Low BS(LBS): BS at 1x10^4^ alone, FOS+LBS, High BS (HBS): BS at 1x10^6^ and FOS+HBS.

Bars sharing same small letter alphabet are not significantly different (p>0.05). Significance of all tests was accepted at *p*=0.05. Data represented as means ±S.E.M. (*n*=3).

**Figure S3**. Gross lesions of infected fish with pathogen, A*. hydrophila*


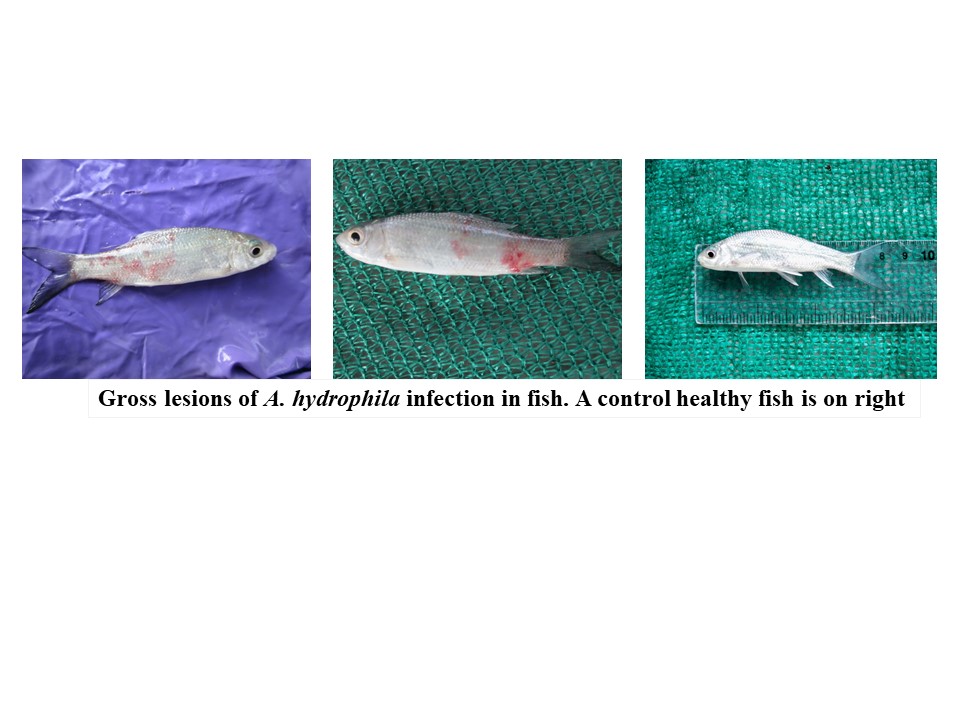

Supplement: Supplementary file 1 — Supplementary Information. [file 41598_2023_38267_MOESM1_ESM.doc]
